# Maternal postpartum blood pressure screening at newborn visits and its impact on postpartum care utilization

**DOI:** 10.1002/pmf2.70111

**Published:** 2025-09-16

**Authors:** Paige M. Anderson, Amanda Krauss, Brianna Blake, Ronit Kar, Julia Pantalone, Adriana Phillips, Gysella B. Muniz, Jacqueline Saladino, Malamo E. Countouris, Alisse Hauspurg

**Affiliations:** ^1^ University of Pittsburgh Medical Center Magee‐Womens Hospital Pittsburgh Pennsylvania USA; ^2^ University of Pittsburgh Medical Center Children's Hospital of Pittsburgh Pittsburgh Pennsylvania USA; ^3^ University of Pittsburgh School of Medicine Pittsburgh Pennsylvania USA; ^4^ Magee‐Womens Research Institute University of Pittsburgh School of Medicine Pittsburgh Pennsylvania USA; ^5^ University of Pittsburgh Medical Center Heart and Vascular Institute Pittsburgh Pennsylvania USA; ^6^ Present address: Department of Obstetrics and Gynecology University of North Carolina Chapel Hill School of Medicine Chapel Hill North Carolina USA; ^7^ Present address: University of Michigan Congenital Heart Center Ann Arbor Michigan USA; ^8^ Present address: Women & Infants Hospital of Rhode Island Alpert Medical School of Brown University Providence Rhode Island USA

**Keywords:** blood pressure, hypertension, maternal‐infant care, postpartum, pregnancy, quality improvement, well‐child care

## Abstract

**Background:**

To assess the impact of a maternal cardiovascular (CV) and blood pressure (BP) screening and standardized referral protocol at newborn visits on care utilization in the first 12 weeks postpartum.

**Methods:**

This prospective quality improvement initiative obtained maternal vital signs and CV symptoms at a pediatric academic practice. Elevated BP (≥140/90 mmHg) triggered enrollment into a remote BP management program, on‐call Maternal‐Fetal Medicine or Cardiology physician consultation, and/or emergency department (ED) referral. Unmatched control group were postpartum individuals of newborns seen on non‐screening days in the same clinic. Outcomes included postpartum visit attendance, ED visits, readmissions, new postpartum hypertension (HTN) diagnosis, remote BP monitoring program enrollment, and postpartum antihypertensive initiation. Multivariable logistic regression was used to calculate odds ratios for postpartum care utilization outcomes and adjusted for demographic differences between groups (parity, BMI, gestational age, smoking) and other covariates related to postpartum care utilization (age, insurance, preexisting HTN). Interaction analyses tested effect modification by preexisting HTN status, with predictive margins calculated when significant using chi‐square tests.

**Results:**

We included 132 screened individuals and 248 controls (77.9% non‐White, 76.3% publicly insured). Among those screened, 34.1% (*n* = 45) had elevated BP, with 38.7% (*n* = 17) having no prior HTN diagnosis. Among screened participants with known HTN (*n* = 45), 62.2% (*n* = 28) had elevated BP at screening. The intervention group had significantly higher rates of newly diagnosed postpartum HTN (20.7% vs. 8.2%, *p* < 0.01). After adjustment, the intervention had no effect on postpartum visit attendance (OR 1.05, 95% CI 0.62–1.78), but reduced the odds of ED visits (OR 0.52, 95% CI 0.29–0.95) and readmissions (OR 0.31, 95% CI 0.11–0.95). For those with preexisting HTN, acute care utilization was similar between groups (ED visits—χ^2^ = 0.56, *p* = 0.45; readmissions—χ^2^ = 0.18, *p* = 0.66).

**Conclusion:**

This intervention detected more postpartum HTN while being associated with decreased odds of ED visits and readmissions. Acute care usage was similar for those with existing HTN, despite high rates of uncontrolled HTN at screening. This suggests maternal BP screening at newborn visits paired with remote BP monitoring may improve our postpartum patient outreach and be more feasible than traditional care while reducing acute care needs.

## 1 BACKGROUND

Maternal morbidity and mortality extend beyond pregnancy and immediately postpartum, with 47% mortality occurring 7+ days postpartum [1, 2]. Hypertension (HTN) and cardiovascular disease (CVD) are leading causes of postpartum complications, including emergency department (ED) visits and readmissions [1, 2, 3, 4]. For example, HTN accounts for approximately 25% of all postpartum ED visits, which complicate approximately 3%–4% of all births. Similarly, readmission rates among those with peripartum HTN are almost 10 times that of baseline rates (8%–9% vs. 1%–2%) [5, 6, 7].

While many postpartum hypertensive complications occur in individuals with a prior HTN diagnosis, studies report the prevalence of new‐onset postpartum HTN (or de novo postpartum hypertension) may range from 5.7% to 8.2% [8, 9, 10]. Individuals diagnosed with new postpartum HTN may face higher morbidity rates, which could potentially be mitigated through earlier postpartum care and detection [11].

Effectively reaching patients for postpartum care remains a significant challenge despite increased emphasis on comprehensive care, with postpartum attendance rates ranging from 24.9% to 96.5% (mean 72.1%) [12]. Those who are younger, non‐White, and have state‐funded health insurance, are less likely to attend postpartum visits and more likely to experience maternal morbidity [1, 13, 14].

Despite low postpartum visit attendance, most birthing parents attend well‐child care (WCC), which has up to 90% attendance in the first two months of life [15, 16]. This presents an opportunity to integrate postpartum care into pediatric settings to improve reach and mitigate care disparities [17].

The most common example of maternal postpartum care at pediatric visits is maternal depression screening, which is now recommended by the American Academy of Pediatrics (AAP) and required by many state Medicaid programs [18, 19, 20, 21]. According to an AAP survey, 83.2% of pediatricians in 2019 reported implementing some form of postpartum depression screening protocol [22]. Other interventions like contraception counseling at WCC have shown feasibility and acceptability to patients as well [23].

Given the significance of HTN‐related postpartum morbidity, low traditional postpartum care participation, and successful implementation of other maternal screenings at WCC, we implemented a novel maternal CV symptom and blood pressure (BP) screening and standardized referral protocol at newborn appointments [24]. This study aims to assess the intervention's impact on postpartum care utilization and HTN outcomes during the first 12 weeks postpartum.

## 2 METHODS

### 2.1 Study design and setting

This prospective quasi‐experimental study evaluated a maternal CV symptom and BP screening quality improvement initiative at an urban academic pediatric care center's newborn clinic serving infants in their first month of life from June 2021 to August 2022. The clinic serves the surrounding urban community and is located within walking distance of the corresponding delivery hospital. This study expanded upon our previously published pilot work that included 72 participants who underwent the screening at a median of 9 days (interquartile range 5–15) postpartum and demonstrated feasibility [24]. This study was approved by the University of Pittsburgh's Institutional Review Board (STUDY 21040187) as an exempt study and by UPMC's Quality Improvement Review Committee.

### 2.2 Participant selection

As previously described in detail in our pilot study, the screening intervention was offered to all postpartum individuals who attended their newborn's visit on certain weekdays when adequate staffing was available [24]. In the current study, we retrospectively identified an unmatched control group comprised of postpartum individuals with newborns who attended visits at the same clinic, from office schedule rosters with delivery dates within 7 days of those in the intervention group, on weekdays when the screening was not offered to reduce potential selection bias by avoiding comparison of those who declined screening. We aimed for approximately a 2:1 control‐to‐intervention ratio to improve the study's statistical power. People were excluded if they did not receive their prenatal and/or delivery care within the same hospital system as the newborn clinic to try to reduce incomplete data collection from the electronic medical record (EMR).

### 2.3 Intervention

The screening protocol employed in this study was identical to that used in the pilot study [24]. First, participants were given a survey administered through University of Pittsburgh's Clinical and Translational Science Institute's REDCap about whether they had attended or scheduled a postpartum visit, HTN history, current antihypertensive medications, and CV symptoms adapted from the California Cardiovascular Toolkit (see the Supporting Information) [25]. Participants then had their vital signs taken with a validated A&D automatic cuff and a pulse oximeter by a clinic staff or study team member during the visit prior to seeing the provider while in the seated position after at least 5 min of rest. We assessed systolic blood pressure (SBP), diastolic blood pressure (DBP), heart rate, and oxygen saturation. Elevated BP was defined as: SBP ≥ 140 mmHg or DBP ≥ 90 mmHg. A positive screen, defined as an elevated BP or ≥ 2 cardiovascular symptoms, triggered the pediatric provider to use a standardized referral protocol which included enrolling the patient in the institution's remote BP management program, contacting an on‐call Maternal Fetal Medicine or Cardiology physician for guidance, and/or ED referral based on severity (see the Supporting Information for algorithm). Recommendations from on‐call providers included remote postpartum BP management program enrollment, refilling or increasing antihypertensive medications, encouraging short interval follow‐up with a person's prenatal or primary care provider, or referring to the ED.

As per our standardized referral algorithm, eligible people were enrolled in our institution's remote BP monitoring program and given a BP device on the same day in the pediatrician's office. This remote postpartum BP monitoring program has been ongoing since 2018 and has been detailed previously [26]. In brief, the program enrolls postpartum participants who have a diagnosis of chronic or pregnancy‐related HTN, speak English, Spanish, or Portuguese, and have a phone with texting capabilities. Participants are equipped with an automatic A&D BP monitor and instructed to submit readings via a text‐based application daily for the first two weeks, and then three to five times a week until 6 weeks postpartum. Antihypertensive medications were prescribed and adjusted by call center physicians based on the reported readings. Enrollees could also report symptoms via the application and receive advice on seeking in‐person care. All data from the program were uploaded into a person's EMR weekly. This remote monitoring BP program takes the place of in‐person BP checks for those enrolled.

### 2.4 Data collection

Maternal characteristics and perinatal details were obtained from the Steve N. Caritis Magee Obstetrics Maternal & Infant (MOMI) Database and Biobank. This database was established in 1995 from all individuals who have delivered at our institution's hospital through the present. It includes information on over 500 variables for maternal, fetal, and neonatal characteristics obtained from admitting services, International Classification of Diseases, Tenth Revision (ICD‐10) codes, electronic medical record abstraction, electronic birth records, and ultrasound. It is populated in real time as a research database, with dedicated data administrators who review, validate, and store the data collected. Postpartum care utilization and outcome data were manually abstracted from the EMR into REDCap, with 20% of records abstracted independently by two different abstractors and compared to ensure accuracy.

The primary outcomes included postpartum care utilization, encompassing postpartum visits, ED visits, and readmissions within the first 12 weeks postpartum. Any visit with an obstetrician, midwife, or primary care provider that addressed postpartum care was included as a postpartum visit. All ED visits and readmissions that were available for review within the EMR within the first 12 weeks postpartum were captured and recorded.

Additional HTN outcomes also assessed included: new diagnosis of postpartum HTN or elevated BP (SBP ≥ 140 or DBP ≥ 90) within the first 12 weeks postpartum after discharge from delivery, including elevated readings at the time of this intervention, enrollment ± participation in our institution's remote BP monitoring program, and new postpartum antihypertensive initiation. Enrollment in the remote BP monitoring program was defined as documentation in the EMR that the program reached out to the patient and participation was defined as a patient having uploaded at least one BP to the program.

### 2.5 Statistical analysis

Statistical analyses were performed using StataNow 18.5 BE software (StataCorp LLC). For univariate analysis, data were assessed for missingness, and missing data were excluded. Categorical variables were summarized using frequencies and percentages. Continuous variables were assessed for normality; normally distributed variables were presented as mean and standard deviation (SD). Non‐normally distributed variables were presented as median and interquartile range (IQR). Variables were then stratified by exposure and proportions were compared using chi‐square or Fisher's exact tests while continuous measures were compared using *t*‐tests and Mann–Whitney U tests for non‐normally distributed variables.

Finally, multivariable logistic regression was used to calculate odds ratios for each outcome among the exposed and unexposed groups, controlling for variables that were unevenly distributed among the groups at baseline and pre‐defined covariates associated with postpartum care utilization, including diagnosis of HTN by delivery discharge, mode of delivery, insurance type, and age, as established in the literature [13, 27].

We also performed an interaction analysis to evaluate whether the presence of an HTN diagnosis (either chronic or pregnancy‐related) at discharge from delivery resulted in effect modification for the association between the intervention and primary outcome. When the interaction term was statistically significant, a predictive margins analysis was conducted to calculate predicted probabilities of postpartum care utilization, and interaction effects were evaluated using chi‐square tests.

Additionally, a subgroup analysis was performed comparing those with de novo postpartum HTN in each group using chi‐square or Fisher's exact tests. Significance was defined a priori as *p* < 0.05 or 95% confidence interval (CI) excluding null values.

## 3 RESULTS

As shown in Table 1, the study included 380 participants (132 intervention, 248 control) with 77.9% non‐White and 76.3% publicly insured. Hypertensive disorders affected 27.1% of participants, with 19.5% having pregnancy‐related HTN and 7.6% having chronic HTN. The intervention group had significantly higher BMI, parity, gestational age at delivery, and smoking rates.

**TABLE 1:** Demographics comparison.

	Intervention group (n = 132)	Control group (n = 248)	*p* value
Age, mean (SD)	27.50 (6.11)	27.58 (5.77)	0.90
Race, n (%)			
White	24 (18.18%)	60 (22.11%)	0.09
Black	99 (75.00%)	154 (62.10%)	
Asian	3 (2.27%)	16 (6.45%)	
Other^a^	1 (0.76%)	6 (2.42%)	
Unknown^b^	5 (3.79%)	12 (4.84%)	
Pre‐pregnancy body mass index, median (IQR)	29.02 (23.13–35.49)	27.42 (22.50–31.47)	< 0.01
Insurance type, n (%)			
Medicaid	102 (77.27%)	188 (75.8%)	0.23
Private	24 (18.18%)	49 (19.8%)	
Self‐pay	4 (3.03%)	11 (4.4%)	
Unknown^a^	2 (1.52%)	0 (0%)	
Marital status, n (%)			
Single	109 (82.58%)	181 (72.98%)	0.10
Married	16 (12.12%)	51 (20.57%)	
Unknown^a^	7 (5.3%)	16 (6.45%)	
Parity, mean (SD)	2.488 (1.48)	2.185 (1.34)	0.048
Gestational age at delivery, mean (SD)	38.79 (1.27)	38.43 (1.90)	0.047
Birth weight, mean (SD)	3,128.98 (481.87)	3,021.12 (544.72)	0.06
Cesarean delivery, n (%)	36 (27.27%)	64 (25.81%)	0.76
Twin pregnancy, n (%)	4 (3.03%)	5 (2.02%)	0.55
Pregnancy‐related hypertension, n (%)	26 (19.70%)	48 (19.40%)	0.94
Chronic hypertension, n (%)	13 (9.80%)	16 (6.50%)	0.24
On antihypertensives at delivery discharge, n (%)	9 (20.00%)	18 (27.7%)	0.36
Diabetes in pregnancy, n (%)	14 (10.60%)	24 (9.68%)	0.10
Smoking status, n (%)			
Never smoker	81 (61.36%)	179 (72.18%)	< 0.01
Former smoker	31 (23.48%)	26 (10.48%)	
Current smoker	15 (11.36%)	38 (15.32%)	
Unknown	5 (3.79%)	5 (2.02%)	

^a^“Other” encompasses: Native Hawaiian or other Pacific Islander, American Indian or Alaska Native, and/or Other not specified as selected in the medical record race/ethnicity category selection category.

^b^“Unknown” was only displayed when there was missing data or declined was an option for a categorical variable, missing data from continuous variables was excluded from analysis.

**FIGURE 1:**
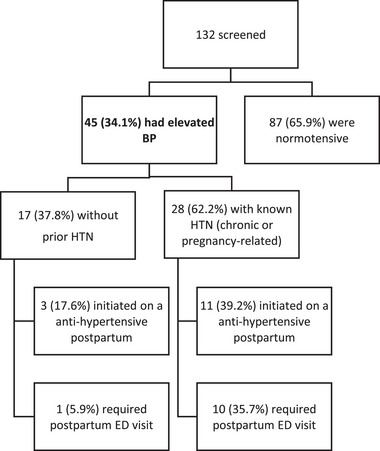
Maternal postpartum BP screening results at newborn visits.

As displayed in Figure 1, among screened participants, 34.1% (*n* = 45) had elevated BP, with 62.2% (*n* = 28) of those with known hypertensive disorders and 37.8% (*n* = 17) with no prior HTN diagnosis showing elevated readings. Of those with elevated BP, 31.1% (*n* = 14) required initiation of antihypertensive medications postpartum, and 24.4% (*n* = 11) required acute care (ED visit and/or readmission).

There were similar rates between groups in postpartum visit attendance (65.0% intervention vs. 67.5% control), ED visits (23.3% vs. 26.5%), and readmissions (10.0% vs. 9.6%). Among all participants who had an ED visit, 32.6% were HTN‐related, and among those with readmissions, 44.8% were HTN‐related. Those with an HTN diagnosis (including chronic and pregnancy‐related) had similar rates of remote BP monitoring enrollment (63.5% vs. 62.7%) and participation once enrolled (85.0% vs. 86.8%) and postpartum antihypertensive initiation (31.4% vs. 25.8%). In multivariable analyses, we took a stepwise approach (Table 2). Unadjusted analysis showed no differences in postpartum care utilization. After adjusting for baseline demographic differences (Model 1) and additional known predictors of care utilization (Model 2), the intervention was not associated with differences in postpartum visit attendance (Model 2: aOR 1.05, 95% CI 0.62–1.78) but associated with significantly reduced odds of ED visits (aOR 0.52, 95% CI 0.29–0.95), and readmissions (aOR 0.31, 95% CI 0.11–0.95) in both models.

**TABLE 2:** Logistic regression models for postpartum care utilization.

OUTCOME	UNADJUSTED OR (REF = CONTROL)	MODEL 1 ADJUSTED OR (REF = CONTROL)^a^	MODEL 2 ADJUSTED OR (REF = CONTROL)^b^
POSTPARTUM VISIT, or (95% CI)	1.01 (0.65–1.57)	1.03 (0.63–1.69)	1.05 (0.62–1.78)
ED VISIT, or (95% CI)	0.67 (0.39–1.13)	0.51 (0.28–0.92)	0.52 (0.29–0.95)
READMISSION, or (95% CI)	0.70 (0.30–1.62)	0.31 (0.11–0.92)	0.31 (0.11–0.95)

^a^ Adjusted model is adjusted for pre‐pregnancy BMI, parity, gestational age at delivery, and smoking status.

^b^ Adjusted model is adjusted for pre‐pregnancy BMI, parity, gestational age at delivery, smoking status, age, delivery type, insurance status, and hypertension diagnosis at delivery discharge.

**FIGURE 2:**
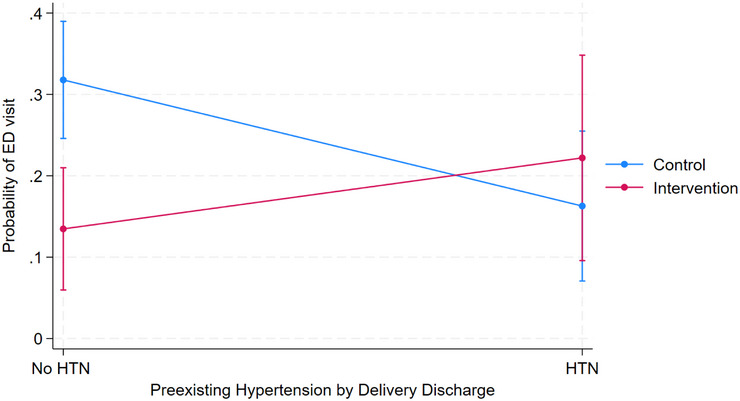
Differential screening effect on ED visit probability by preexisting hypertension status.

**FIGURE 3:**
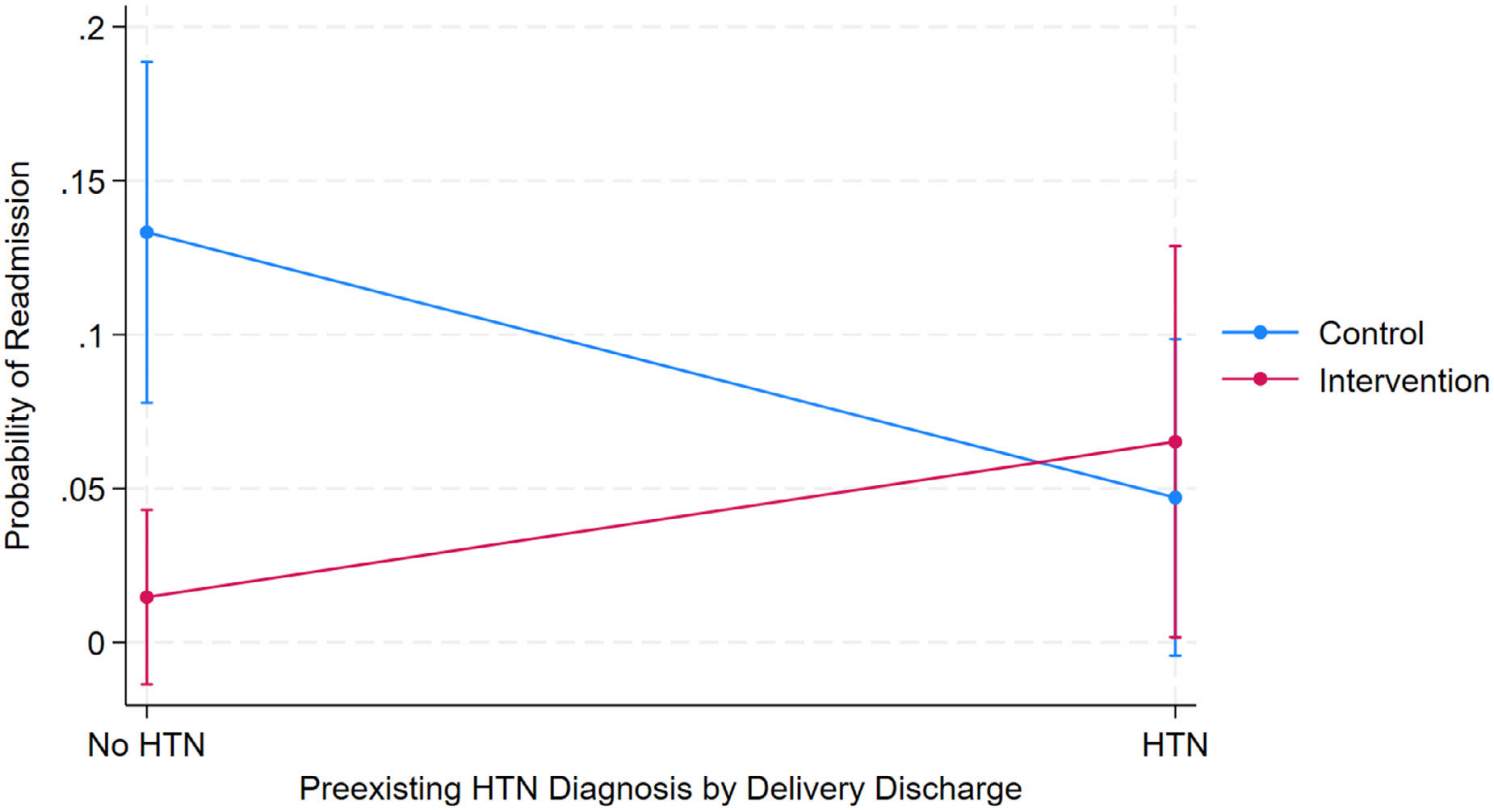
Differential screening effect on readmission visit probability by preexisting hypertension status.

Interaction analysis revealed significant effect modification by preexisting HTN status for ED visits (*p* = 0.02) and readmissions (*p* = 0.03), but not for postpartum visits (*p* = 0.49). As shown in Figures 2 and 3, among participants without preexisting HTN, the intervention significantly reduced predicted probability of ED visits (0.14 vs. 0.32, *p* < 0.01) and readmissions (0.01 vs. 0.13, *p* < 0.01), while showing no significant differences for those with preexisting HTN. The overall interaction effect was statistically significant for both outcomes (*p* < 0.01), indicating that preexisting HTN influenced the impact of the intervention.

The intervention group had significantly higher rates of new HTN postpartum diagnosis (20.7% vs. 8.2%, *p* < 0.01), with 94.4% identified through this screening. Despite this, intervention participants with new HTN had lower rates of ED visits (11.1% vs. 73.3%, *p* < 0.01) and readmissions (0.0% vs. 33.3%, *p* < 0.01). Intervention participants had lower rates of enrollment in remote BP monitoring (27.8% vs. 66.7%, *p* = 0.03), but among those enrolled, participation rates were similar (80% vs. 90%, *p* = 0.59). There was no significant difference in antihypertensive initiation (22.2% vs. 46.7%, *p* = 0.14) (Table 3).

**TABLE 3:** Comparison of care utilization and HTN outcomes among individuals with new postpartum HTN.

OUTCOME	INTERVENTION (*n* = 18)	CONTROL (n = 15)	*p* Value
New postpartum HTN, % (*N*)	20.7% (18/87)	8.2% (15/183)	< 0.01
ED visits, % (*N*)	11.1% (2/18)	73.3% (11/15)	< 0.01
Readmissions, % (*N*)	0.0% (0/18)	33.3% (5/15)	< 0.01
Postpartum visit, % (*N*)	83.3% (15/18)	73.3% (11/15)	0.48
Antihypertensive initiation, % (*N*)	22.2% (4/18)	46.7% (7/15)	0.14
Enrolment in remote BP monitoring, % (*N*)	27.8% (5/18)	66.7% (10/15)	0.03
Participation in remote BP monitoring, % (*N*)	80.0% (4/5)	90.0% (9/10)	0.59

## 4 DISCUSSION

Building upon our pilot study, we demonstrated that maternal BP screening at newborn visits combined with remote BP monitoring was associated not only with increased identification of postpartum HTN but also with reduced acute care utilization. Specifically, our intervention detected significantly more new postpartum HTN compared to standard care—20.7% in the intervention group versus 8.2% in controls. The control group rate aligns with prior literature, reinforcing the potential value of universal rather than risk‐based screening [8, 9, 10]. Furthermore, our findings were comparable to our pilot results, which showed 43.1% had elevated BP at screening, with a rate of 24.5% of new postpartum HTN, providing consistent evidence of this screening's utility. This study moves beyond our pilot's proof of concept to demonstrate that maternal BP screening in pediatric settings represents an effective approach to addressing the critical need for earlier postpartum assessment and better reach of postpartum individuals.

Our analysis of healthcare utilization revealed important nuances. While unadjusted rates appeared similar between groups, adjusted models showed a significant association between our intervention and reductions in ED visits and readmissions. Controlling for baseline demographic differences was sufficient to reveal the intervention's protective effect, suggesting that baseline demographic differences were the reason unadjusted results were insignificant. The minimal change between Model 1 and Model 2 further suggests that the intervention's effect was robust and independent of additional clinical risk factors known to influence postpartum care utilization.

The intervention particularly benefited those without preexisting HTN and those with newly diagnosed postpartum HTN, who experienced substantially lower acute care utilization. Additionally, the similar rates of antihypertensive medication initiation between groups for those with new postpartum HTN, despite the dramatic reduction in acute care utilization, suggest two possible interpretations: our screening may be identifying less severe disease earlier in its clinical course and/or our intervention pathways—including immediate provider consultation and remote monitoring enrollment—may be providing superior outpatient management that prevents disease progression requiring emergency intervention. The substantial reduction in acute care needs despite comparable medication requirements supports the latter interpretation, indicating that early detection combined with proactive management may prevent complications that would otherwise necessitate hospital‐based care. This is clinically significant considering that new‐onset postpartum hypertension is associated with substantial morbidity. Previous studies report ED visit and readmission rates ranging from 8% to 66% depending on study methodology and population, with new‐onset cases comprising up to 50% of all postpartum preeclampsia readmissions [8, 9, 28, 29]. Our control group with new postpartum HTN experienced rates of 73.3% ED visits and 33.3% readmissions, which align with the higher end of this range, emphasizing the substantial morbidity associated with this diagnosis.

On the other hand, for participants with preexisting HTN, acute care utilization was similar between groups despite the intervention detecting significant uncontrolled HTN in this population. This stability is notable—despite identifying more cases requiring attention, acute care usage did not increase, suggesting successful outpatient management through our screening program. Alternatively, the comparable rates of remote blood pressure monitoring in both groups among those with preexisting HTN may have reduced the impact of this additional screening, still demonstrating the importance of close postpartum BP monitoring and management for preventing acute care utilization.

Our findings contrast with Amro et al., who reported increased readmission after a postpartum BP screening at well‐child visits—a result they viewed positively as evidence of necessary care for identifying more uncontrolled postpartum HTN [29]. This difference in results likely stems from variations in management algorithms. Both studies referred those with severely elevated BP (SBP ≥160 or DBP ≥110) or those with mildly elevated BP (SBP ≥140 or DBP ≥90) with significant preeclampsia symptoms to the ED [29]. However, our approach differed by providing on‐call maternal care consultations and enrolling patients with asymptomatic mild elevations in remote BP monitoring rather in addition to instructing them to contact their providers [29]. While we cannot definitively separate the effects of the screening from the remote BP monitoring program, the combination appears to facilitate earlier identification and outpatient management of postpartum HTN while reducing avoidable acute hospital‐based care. Given the substantial financial burden associated with postpartum readmissions—estimated at $33,000 per hospitalization—our results suggest that our model may offer a clinically and economically meaningful improvement in postpartum HTN care [30].

Our intervention did not significantly impact postpartum obstetric visit attendance, even among those with HTN. While we hypothesized that providing maternal BP screening and directly inquiring about postpartum visit scheduling would motivate participants to prioritize their own care, this combined approach did not increase visit attendance as expected. This suggests that structural and logistical obstacles to traditional postpartum care remain unaddressed by our screening approach. However, the high rates of participation in the remote BP monitoring program among both groups suggest that alternative care delivery models that prioritize convenience and bring care to the patient may more effectively meet postpartum individuals' needs. These alternative models, including telehealth visits, remote monitoring technologies, including universal offering of postpartum home BP monitoring, text‐based check‐ins, and home visitation programs, can reduce care engagement barriers, particularly benefiting those from systematically marginalized populations with increased risk of barriers to traditional care. However, universal home monitoring may still have patient engagement limitations, and pediatric visit screening can offer complementary safety net value by leveraging high‐attendance healthcare encounters while also being implementable at places where universal BP monitoring programs cannot be offered due to resource limitations.

Strengths of this study include the substantial expansion from our pilot work, moving beyond feasibility to assess healthcare utilization outcomes. The study's implementation in a busy urban academic practice serving a predominantly non‐White (77.9%) and publicly insured (76.7%) population demonstrates the potential for addressing known disparities in postpartum care. Additionally, the integration with an established remote BP monitoring program allowed for immediate intervention for those identified with elevated blood pressure, potentially contributing to the reduced acute care utilization observed.

There were several limitations to this study. There may be potential selection bias, as we lack data on individuals who declined screening. Our reliance on EMR review may have missed care sought outside our health system. Additionally, the study was limited by sample size and study design. The non‐randomized design of this study makes it challenging to fully account for confounding or contamination effects, as evidenced by baseline differences between intervention and control groups. This limitation could impact our ability to adequately detect differences in more rare events, such as postpartum readmissions.

We lack temporal data on ED visits, readmissions, and remote BP monitoring enrollment timing relative to our intervention, limiting our ability to establish causality and disentangle the effects of screening versus remote monitoring components. We also lack data on staff's adherence to the standardized referral protocol, which could help explain the remote monitoring enrollment discrepancies in our subgroup analysis of those with new postpartum HTN.

Finally, while we intentionally implemented this intervention as a clinic that served a population with a higher baseline HTN risk profile to maximize impact, it may limit generalizability to lower‐risk settings.

## 5 CONCLUSIONS

Maternal hypertension screening, integrated into pediatric care settings with standardized escalation pathways, represents a scalable, patient‐centered approach to improving both postpartum hypertension detection and care. This intervention has the potential to be cost saving by decreasing acute care utilization, particularly among those without previously diagnosed HTN. Future work should focus on implementation science approaches to improve adoption and fidelity of the intervention and to optimize the screening for different pediatric settings, including those with limited resources. Research priorities should include testing various implementation strategies, identifying essential components versus adaptable elements of the screening protocol, developing algorithms for settings without established remote monitoring programs, and evaluating the strategies for sustainability. By addressing these implementation questions, we can move beyond proof‐of‐concept to widespread adoption of postpartum blood pressure screening in pediatric settings.

## CONFLICTS OF INTEREST STATEMENT

The authors declare no conflicts of interest.

## ETHICS STATEMENT

This study was approved by the University of Pittsburgh Institutional Review Board as an exempt study (STUDY 21040187) and the University of Pittsburgh Medical Center Quality Improvement Review Committee.

## Supporting information



Supporting information

## Data Availability

The datasets are available from the corresponding author on reasonable request.
