# Supplementary material for: Maternal postpartum blood pressure screening at newborn visits and its impact on postpartum care utilization
Source: Pregnancy (Hoboken). 2025 Sep 16;1(5):e70111. doi: 10.1002/pmf2.70111 (PMC13344582; doi:10.1002/pmf2.70111)
Supplement: Supplementary file 1 [file PMF2-1-e70111-s001.docx]

Supplemental Materials:
